# Characterization of the complete chloroplast genome of *Leptochilus decurrens* (Polypodiaceae), a least concern folk medicinal fern

**DOI:** 10.1080/23802359.2019.1673243

**Published:** 2019-10-04

**Authors:** Yingjuan Su, Ziqing He, Zhen Wang, Yongfeng Hong, Ting Wang

**Affiliations:** a School of Life Sciences, Sun Yat-sen University, Guangzhou, China;; b Research Institute, Sun Yat-sen University, Shenzhen, China;; c College of Life Sciences, South China Agricultural University, Guangzhou, China

**Keywords:** *Leptochilus decurrens*, chloroplast genome, phylogenetic analysis

## Abstract

*Leptochilus decurrens* is a small tropical fern of Polypodiaceae, but also a least concern folk medicinal fern. Its genome size is 153,753 bp with a pair of 21,664 bp inverted repeat regions (IR), a large single-copy (LSC) region of 84,395 bp and a small single-copy (SSC) region of 26,030 bp. A total of 133 genes were predicted, including 88 protein-coding genes, 37 tRNA genes and eight rRNA genes. The ML analysis reveals that *L. decurrens* is sister to *Leptochilus hemionitideus* forming monophyletic group closely related with polypodiaceous ferns. The work provides vigorous molecular data to further promote phylogenetics of Polypodiaceae.

*Leptochilus decurrens* Blume is a small tropical fern of Polypodiaceae, which is also the most widely ranging species in genus *Leptochilus*, with distribution in southern China, India, Indonesia, Sri Lanka, and Polynesia etc. (Zhang et al. 2013). The species prefers to grow on rocks beside streams in forests with latitude of 100–1800 m. Its rhizome is dorsiventrally flattened, with only scattered strands of sclerenchyma (Zhang et al. 2013). As a least concerned fern (Banerjee et al. 2018), *L. decurrens* is used in Hmong folk medicine to activate blood circulation and relieve pain (Pan et al. 2011). In addition, *Leptochilus* has a very complicated taxonomic history (Copeland 1929), systematic position of which is highly controversial. Especially, as one of microsoroid ferns with *Microsorum* and *Podosorus*, whether or not they should be separated from other polypodiaceous ferns is also disputed (Boonkern and Pollawatn 2002). Hence, sequencing the complete chloroplast genome of *L. decurrens* will provide new approaches to settle these systemic disputes.

We sampled *L. decurrens* from South China Botanical Garden, Chinese Academy of Sciences (23°19′28.21″N, 113°37′47.46″E). Its voucher specimens were deposited in Herbarium of Sun Yat-sen University (SYS; voucher: *SS Liu 20161022*). Total genomic DNA was extracted from fresh leaves using Tiangen Plant Genomic DNA Kit (Tiangen Biotech Co., Beijing, China) and broken into 300 bp fragments. After Illumina paired-end library construction, high-throughput DNA sequencing was conducted on Hiseq 2500 platform (Illumina Inc., San Diego, CA). In total, 11621272 raw reads were obtained. After base quality control using Trimmomatic v0.32 (Bolger et al. 2014), the remaining high-quality reads were used to assemble the chloroplast genome by Velvet v1.2.07 (Zerbino and Birney 2008), which was further filled gaps using PCR. The plastome was annotated using DOGMA (Wyman et al. 2004) and tRNAscan-SE programs (Lowe and Eddy 1997) with manual correction for start and stop codons. The accurate gene boundaries were confirmed by alignment with other chloroplast gene of ferns. We generated a maximum-likelihood (ML) tree through RAxML v.8.0 with 1000 bootstrap replicates (Stamatakis 2014) based on a 14 complete chloroplast sequences aligned using the MAFFT v7.311 (Katoh and Standley 2013) including *Macrothelypteris torresiana* as outgroup.

The complete chloroplast genome of *L. decurrens* (GenBank accession number: MN044573) was determined as 153,753 bp in length, containing a pair of 21,664 bp inverted repeat regions (IR), a large single-copy (LSC) region of 84,395 bp and a small single-copy (SSC) region of 26,030 bp. GC content of LSC, SSC, and IR is 43%, 43%, and 47%, respectively. The genome has 133 genes, involving in 88 protein-coding genes, eight rRNA genes and 37 tRNA genes. Among 17 genes with introns, nine have a single intron (*ndhB, rps16, atpF, rpoC1, petB, petD, ndhA, rpl16,* and *rpl2*), and remaining three possess two introns (*ycf3*, *clpP*, and *rps12*). Four protein-coding genes, five tRNA genes, and four rRNA genes were duplicated within the IRs. The ML analysis reveals that *L. decurrens* is sister to *Leptochilus hemionitideus* forming monophyletic group closely related with polypodiaceous ferns (Figure 1). The study provides powerful resource to facilitate the chloroplast phylogenomics of Polypodiaceae.

**Figure 1.**
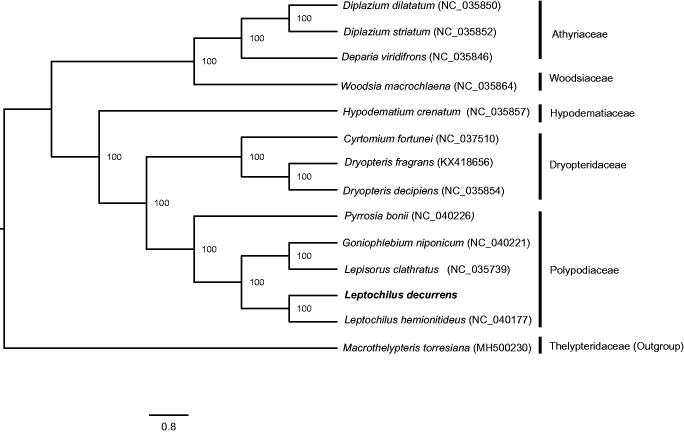
Maximum likelihood phylogenetic tree based on 14 complete chloroplast genome sequences of ferns using RAxML v.8.0 with 1000 bootstrap replicates. Numbers near branches are support values.
